# Icing Detection of Wind Turbine Blades Based on an Improved PP-YOLOE Detection Network

**DOI:** 10.3390/s25206438

**Published:** 2025-10-17

**Authors:** Zhangzhuo Sun, Jiangbo Qian, Ao Liu, Shangyun Yao, Xinzhu Lv, Liwei Shao

**Affiliations:** 1 Yanzhao Electric Power Laboratory, North China Electric Power University, Baoding 071003, China; s13233574080@163.com (Z.S.); qjb@ncepu.edu.cn (J.Q.); 18554300813@163.com (X.L.); 2 Hebei Key Laboratory of Low Carbon and High Efficiency Power Generation Technology, Baoding 071003, China; 3 China National Water Resources & Electric Power Materials & Equipment Group Co., Ltd., Beijing 100040, China; yaooosy@outlook.com; 4 School of Automation, Beijing Institute of Technology, Beijing 100081, China; shaolw@bit.edu.cn

**Keywords:** wind turbine blades, real-time detection, coordinate attention, atrous spatial pyramid pooling, particle swarm algorithm

## Abstract

In cold and highly humid regions, wind turbine blades (WTB) are susceptible to icing, which can have a significant impact on the security and economic operation of turbines. Therefore, precise and prompt icing status detection is pivotal for maintaining wind turbine operational normalcy. In this research, an improved PP-YOLOE network is developed for classifying and detecting the icing state of WTB. First, a dataset of WTB icing is constructed based on a wind tunnel laboratory and expanded to improve the generalization of the model. To enhance feature representation, the network architecture was improved by embedding a coordinate attention (CA) mechanism and integrating atrous spatial pyramid pooling (ASPP) to better capture multi-scale contextual information. Moreover, a key innovation of this work is the novel application of a particle swarm optimization (PSO) algorithm to systematically automate hyperparameter tuning. Through ablation experiments and comparative tests, the improved PP-YOLOE network demonstrates superior overall performance on this dataset, achieving a multiple average precision of 0.94. It surpasses the original model across multiple evaluation metrics, indicating a robust and meaningful enhancement. The improved PP-YOLOE network proposed in this study provides a promising and effective method for WTB icing detection. This work provides a paradigm for applying advanced deep learning techniques to enhance intelligent industrial inspection tasks.

## 1. Introduction

To realize the goal of sustainable development, many countries are striving to promote energy transition. According to the International Renewable Energy Agency (IRENA) [1], in 2022, the global installed capacity of renewable energy generation reached approximately 295 GW, which was an 83% share of the global capacity expansion, with wind energy adding 74.7 GW, accounting for approximately 25% of the total. It is expected that the contribution of wind power generation will increase by 5–30% by 2050 [2]. Cold, high-altitude climates provide around 10% more exploitable wind energy because denser air increases power output [3,4], prompting large wind-farm deployment in China’s “Three-North” region and the Yunnan–Guizhou Plateau [5] as well as in northern Europe and North America [6]. However, the existence of moisture in such environments can pose a higher risk of freezing for wind turbine units, especially exposed components such as WTB and anemometers [7]. Moreover, the rapid development of offshore wind power in recent years has also faced icing issues, as high moisture content and ocean splashing cause WTB freezing at low temperatures [8].

The main hazards of WTB ice cover include variation in blade aerodynamic profile, changes in load distribution, ice throw, and damage to sensing equipment. (1) Icing, especially glaze ice formation, significantly alters the flow field, resulting in reduced aerodynamic performance of the WTB [9]. When the WTB lift coefficient is reduced, it will affect the output power, resulting in economic damage; when the stall of WTB is delayed, the wind turbine is prone to overload, and safety will be affected [10,11]. Gao et al. [12,13,14,15] conducted a series of experimental studies using icing tunnels to investigate the degradation of aerodynamic performance due to ice accumulation through the dynamic process of ice accumulation on surfaces. (2) Uneven load distribution due to icing can exacerbate the fatigue of WTB and even cause them to crack. Afzal et al. [16] proposed that the weight of ice on a WTB can be up to 50% of its weight. Moreover, this uneven distribution induces additional vibration in WTB [17]. (3) The centrifugal force generated by the rotation of the wind turbine can throw ice from the blades, posing a threat to the safety of nearby people, buildings, etc. [18,19]. Tammelin B et al. [20] found that the distance over which ice is thrown from WTB can reach 1.5 times the sum of the turbine height and the rotor diameter. The shedding of bulk ice induces additional vibrations [21,22]. (4) The presence of ice on WTB interferes with sensing equipment, such as anemometers and wind vanes [23]. This affects the control system of the wind turbine, causing the turbine to operate away from optimal conditions. In 2021, heavy snowfall in Texas severely impacted the operation of local wind turbines, resulting in various problems, including pump system failures, internet outages, and heating issues [24]. Hence, it is crucial to develop an appropriate monitoring method to detect icing on the surface of WTB.

Wind turbine condition monitoring becomes increasingly important in wind farm management [25]. This aligns with a broader trend in engineering where dynamic modeling of a physical system’s input and output data is foundational to creating digital twins and enabling intelligent risk assessment for critical infrastructure. For instance, recent studies have developed sophisticated probabilistic models to predict extreme events and assess risks in transportation infrastructures based on monitoring data [26]. The core principles of using sensor-derived data to identify risk states are directly transferable to energy systems, where early and accurate detection of anomalies like icing is paramount. The inspection of WTB can be categorized into offline and online methods [27]. Offline monitoring involves shutting down the units for regular inspections, which is not only expensive but impractical for identifying failures between inspection intervals [28]. Therefore, online condition monitoring technology is more suitable for wind farms with longer inspection intervals [29,30]. The use of computer vision, a real-time detection tool, to detect WTB icing has the following advantages: (1) The ability to detect the icing condition of WTB in the early stages. (2) There is no need to install monitoring equipment on the surface of WTB or inside, which could protect the aerodynamic properties of wind turbines. (3) The operation and maintenance of detection equipment is convenient.

The use of computer vision for the detection of WTB icing is currently divided into two categories: the traditional method, which relies primarily on image preprocessing, machine learning, and edge detection; and the deep learning method, which relies on convolutional neural network-based target detection algorithms. The basic train of thought behind traditional detection is to preprocess images with image denoising, enhancement, correction, etc., and then discriminate the ice-covering condition by using the edge detection algorithm or pixel coordinate judgment. Hu et al. [31] used the above-mentioned preprocessing method to solve issues of low contrast, unclear details, and blurred edges in the data. Subsequently, the edge of icing on WTB was detected using multiscale wavelet analysis, and the thickness of ice on the leading edge of WTB was calculated using pixel coordinates. Similarly, M. Akhloufi et al. [32] and G. Skrimpas et al. [33] all used traditional detection methods to identify WTB icing images by image preprocessing combined with different classification or edge detection algorithms. The aforementioned traditional methods, along with hybrid approaches that integrate conventional techniques with the strengths of deep learning, continue to exhibit substantial application value and competitiveness, primarily attributed to their streamlined design and robust interpretability. In specific scenarios—such as environments with stable lighting, simple backgrounds, or constrained computing resources—the traditional methods referenced above are feasible for detecting the icing state of wind turbine blades (WTB). Nevertheless, the detection process of these methods excessively hinges on prior information that only remains valid within specific environmental contexts. This over-reliance not only leads to insufficient efficiency but also renders them incapable of meeting the demands of real-time monitoring. As a consequence, such detection methods are deficient in universal applicability. Furthermore, enhancing model robustness in complex operational environments is a key research focus; some studies even show that deliberately introducing ‘positive-incentive noise’ can paradoxically improve the diagnostic accuracy of AI models, challenging traditional denoising paradigms [34]. Simultaneously, as models become more complex, their ‘black-box’ nature poses a significant challenge for deployment in safety-critical systems, making the pursuit of interpretable AI methods that can provide physically meaningful results a vital research avenue [35].

With the rapid development of deep learning, it has been widely used in the field of WTB image recognition, but most of the current research using deep learning to detect WTB focuses on the detection of surface damage [36,37,38], and fewer studies have focused on WTB ice cover detection. Markus et al. [39] installed a camera on the nacelle of a wind turbine to record the ice on the blades. They compared various convolutional neural network models and determined that the VGG19 model exhibited the highest level of performance. Kemal H et al. [24] trained several network models to classify WTB icing and demonstrated their reliability with the category activation graph method. Finally, the accuracy of the proposed model is verified by comparing it with the best model using U-Net. In comparison to previous studies employing general classification models such as VGG19, ResNet-50, and Inception-V3, YOLO and its improved versions exhibit notable advantages in model design, performance, application scenarios, as well as efficiency and real-time capabilities. These models are typically designed to concurrently identify objects within images and localize their positions. However, in comparison to the currently widely used target detection model, it continues to encounter the challenge of achieving a balance between precise positioning and swift detection.

Accordingly, to achieve real-time detection of WTB icing thickness, this anchor-free method has fast inference speed while maintaining high accuracy, making it suitable for real-time object detection tasks. This paper proposes an improved PP-YOLOE network and realizes fast and accurate recognition on the dataset constructed by the wind tunnel laboratory. The main contributions of this paper include the following three points:

1. A wind-tunnel–based dataset of wind-turbine-blade icing states is established, filling a data-resource gap in the field and providing a solid foundation for subsequent research.
2. An improved PP-YOLOE network is developed with multiple innovations over the original version, including embedding a Coordinate Attention (CA) mechanism in the CSPRes backbone and introducing an Atrous Spatial Pyramid Pooling (ASPP) module into the CSPBlock of the PANet structure; these enhancements markedly strengthen the model’s ability to detect blade icing.
3. Particle Swarm Optimization (PSO) is applied for the first time to tune the model’s parameters, further boosting detection performance and constituting another key innovation of the study.

## 2. Model and Methods

**Figure 1:**
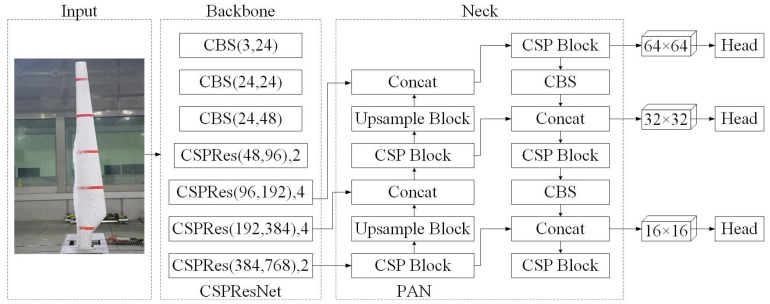
The structure of the PP-YOLOE network.

To improve the speed and accuracy of the measurement of ice cover thickness detection on WTB, this paper introduces the base model PP-YOLOE. This is an ‘evolved’ version of the YOLO (You Only Look Once) series of models, and it is an industrial target detector with high performance and easy deployment [40]. The PP-YOLOE network structure is divided into three key components: backbone, neck, and head. Backbone uses the CSPRes network to extract features. Neck employs the PANet structure for multiscale fusion. The head completes the classification and regression tasks. Figure 1 shows the PP-YOLOE network structure as well as core module components. In the backbone of Figure 1, the first parameter denotes the change in the number of channels, while the second parameter represents the number of ResBlocks in the CSPRes structure.

To improve the detection accuracy of WTB icing state, not only have CA and ASPP been introduced to improve detection performance but an innovative approach to PSO has also been used to optimize the model parameters [41,42]. These improvements have led to an increase in the performance of the model, which can identify the ice cover thickness condition on WTB with higher accuracy.

### 2.1. Coordinate Attention-Based Model of Ice Thickness Measurement Y-Att-Net

The ice cover thickness measurement model Y-Att-Net was constructed by introducing an attention mechanism based on PP-YOLOE, which is a module with a higher ability to capture ice cover features.

**Figure 2:**
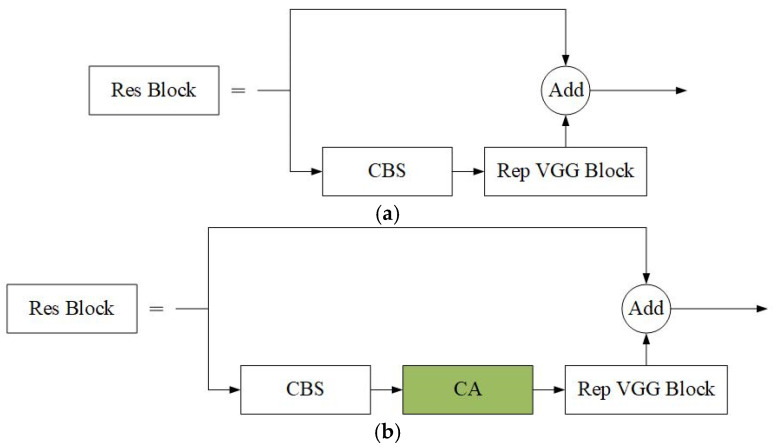
Structure of ResBlock before and after embedding the coordinate attention module. (**a**) The structure of ResBlock in the original network. (**b**) Embedding coordinate attention to the ResBlock.

In traditional backbone networks, feature extraction is mainly based on local relationships, which means that the network can only consider local regions in the input data and cannot effectively capture global or directional position information. As the depth of the network increases, model performance may decrease because it becomes more difficult to extract useful features using traditional methods. To address this problem, an attention mechanism was used that considers directional location information and focuses on channel information. Specifically, coordinate attention [43] was embedded into the Res Block module of the backbone network, a common residual network component used to solve the gradient vanishing or gradient explosion problem in deep networks, and the structure of the Res Block is shown in Figure 2a. The Coordinate Attention (CA) module is embedded into the Res Block module, specifically after the CBS and before the Rep VGG Block, as shown in Figure 2b. The incorporation of the CA module enhances the attention mechanism and the utilization of positional information, effectively improving the perception of ice-edge and texture features while maintaining the model’s lightweight nature.

**Figure 3:**
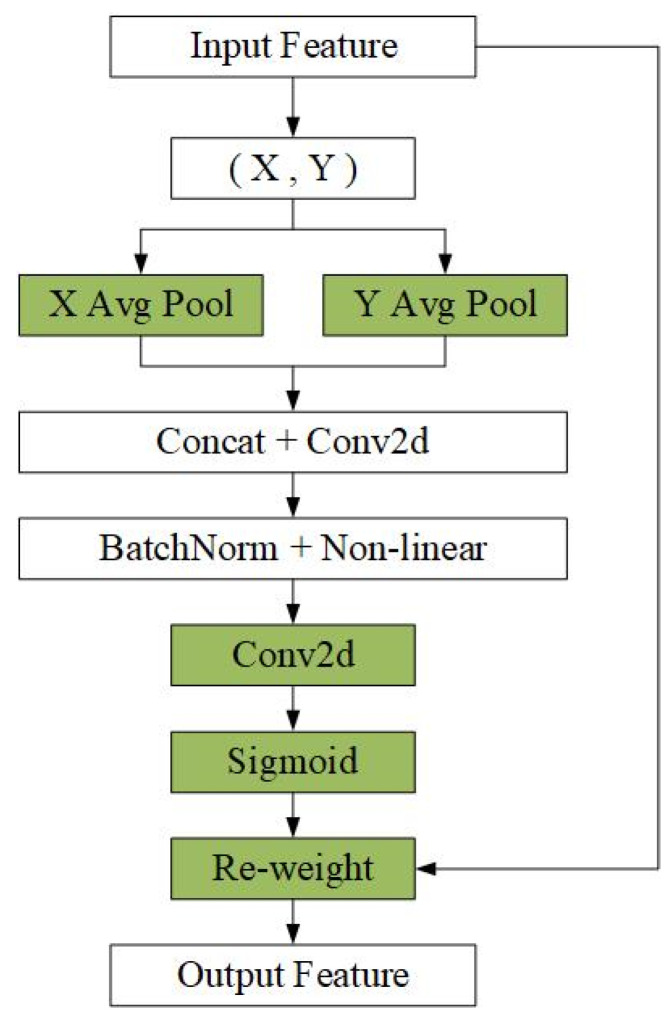
Coordinate attention generation process.

Coordinate attention encodes channels and establishes dependencies over long distances through accurate positional information. The goal of coordinate attention is to encode accurate positional information to establish dependencies between channels and distances during feature extraction. The introduction of coordinate information enables the model to better utilize position information to establish dependencies between channels, thus improving the localization and recognition performance. To better understand the coordinate attention mechanism, Figure 3 specifically depicts the process of generating coordinate attention.

Given input X, each channel encodes a dimension along the horizontal and vertical axes using (H, 1) and (1, W), respectively. Thus, the output of the c-th channel with height h and the output of the c-th channel with width w can be expressed as

(1) $$\begin{array}{c} z_{c}^{h}\left(h\right)=\frac{1}{W}\sum_{0\leq i\leq W}x_{c}(h,i)\\ z_{c}^{w}\left(w\right)=\frac{1}{H}\sum_{0\leq j\leq H}x_{c}(j,w) \end{array}$$

The aggregated feature maps obtained from the c-th channel above in the height and width formulas are spliced, and the spliced features are next fed into a common 1×1 convolutional transform function $F_{1}$ to obtain the intermediate feature mapping f:

(2) $$f=\delta \left(F_{1}\left(\left[z^{h},z^{w}\right]\right)\right)$$

where $\left[\cdot ,\cdot \right]$ denotes the concatenation operation along the spatial dimension, *δ* is a non-linear activation function (specifically, a ReLU function in our implementation), and $f\in R^{C/r\times (H+W)}$ is an intermediate feature mapping encoding spatial information in the horizontal and vertical directions. Along the spatial dimension, f is split into two different tensors $f^{h}\in R^{C/r\times H}$ and $f^{w}\in R^{C/r\times W}$. Using two 1×1 convolutions $F_{h}$ and $F_{w}$, $f^{h}$ and $f^{w}$ are converted into two tensors with the same number of input X channels. The equation can be expressed as

(3) $$\begin{array}{c} g^{h}=\sigma \left(F_{h}\left(f^{h}\right)\right)\\ g^{w}=\sigma \left(F_{w}\left(f^{w}\right)\right) \end{array}$$

where σ is the sigmoid activation function, which scales the output to a range of [0, 1]. The resulting $g^{h}$ and $g^{w}$ serve as the attention weights for the horizontal and vertical directions, respectively. The final output of the coordinate attention module is then computed as

(4) $$y_{c}\left(i,j\right)=x_{c}\left(i,j\right)\times g_{c}^{h}\left(i\right)\times g_{c}^{w}\left(j\right)$$

where $x_{c}\left(i,j\right)$ is the basic feature input at $\left(i,j\right)$, $y_{c}\left(i,j\right)$ is the output of the coordinate attention module at $\left(i,j\right)$.

### 2.2. Atrous Spatial Pyramid Pooling-Based Model of Ice Thickness Measurement SP-Y-Net

The SP-Y-Net model is created by combining Y-Att-Net with the ASPP [44] structure. This model further improves ice thickness detection performance through a wider range of sensor fields and feature extraction, allowing more accurate identification of ice cover conditions at different scales.

**Figure 4:**
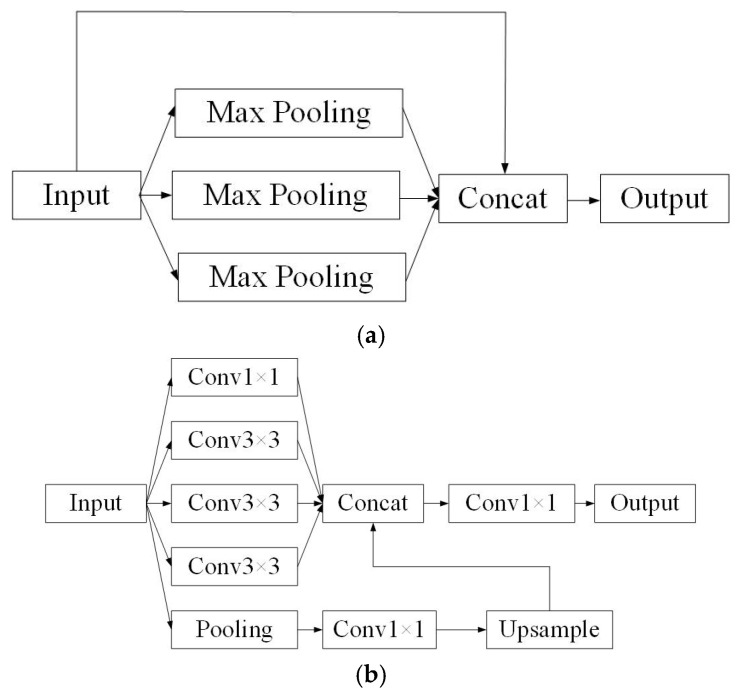
Schematic diagram of the space pyramid pooling structure before and after improvement. (**a**) The SPP structure. (**b**) The ASPP structure.

In the neck of PP-YOLOE, the SPP structure serves to extract information from different receptive fields. As illustrated in Figure 4a, it achieves this by employing multiple parallel max-pooling layers, each with a different kernel size. This allows the model to capture feature information at various scales. However, we argue that it still cannot adequately capture the relations in global and local contexts when compared to our proposed alternative. The theoretical advantage of replacing SPP with ASPP lies in how they capture multi-scale information. SPP utilizes max-pooling with different kernel sizes, which aggressively down-samples features and can lead to a loss of fine-grained spatial detail. In contrast, ASPP employs atrous (dilated) convolutions with varying dilation rates. This allows the model to probe features at multiple scales and capture a wider context without reducing spatial resolution. For WTB icing detection, this is particularly beneficial: a small dilation rate can focus on the texture of fine ice crystals, while a large dilation rate can perceive the overall shape and extent of the ice coverage along the blade’s edge. This richer, multi-scale contextual information, gathered without sacrificing spatial detail, theoretically leads to more robust feature representation and, consequently, superior detection performance compared to the SPP module. By applying different expansion rate expansion factors to the input features, the convolution operation is completed, multiple feature maps and the feature maps of the pooled input features are merged, and finally, the fused features are output using a 1×1 convolution. The working process of ASPP is depicted in Figure 4b.

PP-YOLOE networks utilize SPP solely in the first CSPBlock module of the multiscale-structured PANet and not in any other CSPBlock modules. In contrast, SPP is replaced not only in the first CSPBlock module of the multiscale-structured PANet with ASPP but also in integrating ASPP into other CSPBlock modules.

**Figure 5:**
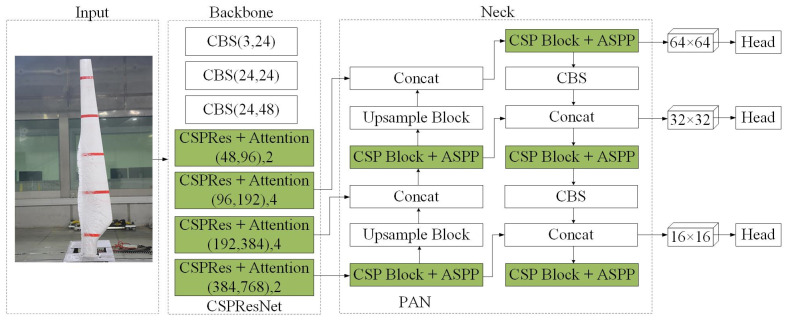
The structure of SP-Y-Net.

From the previously discussed stage onwards, this paper presents two improvements to the PP-YOLOE network structure, as shown in Figure 5. First, the attention mechanism is integrated into the CSPRes module of the backbone network. Second, ASPP replaces SPP in the initial CSPBlock module of the intermediate layer network and is also incorporated into other CSPBlock modules.

### 2.3. PSO Algorithm-Based Model of Ice Thickness Measurement PSO-Opti-Net

To further enhance the performance of the model, we introduce a parameter optimization phase using the Particle Swarm Optimization (PSO) algorithm, resulting in the final PSO-Opti-Net model. It is important to clarify that our innovation does not lie in modifying the canonical PSO algorithm itself, but rather in its novel application to automate the hyperparameter tuning process for a WTB icing detection network. This approach systematically explores the complex parameter space (e.g., learning rate, batch size) to identify a more optimal configuration than what is typically achievable through manual or grid-search methods, thereby constituting a key contribution of our methodology. The performance of the model depends greatly on the choice of parameters, and to achieve the best combination of parameters, this paper employs PSO, the core idea of which is to build a particle swarm, where each particle represents a set of parameters of the SP-Y-Net model. These hyperparameters, which govern the training process, include the initial learning rate, the batch size, and the momentum for the Adam optimizer. The PSO continually updates the velocity and position of the particles to find parameter combinations with better fitness values. This process is accomplished through many iterations, the particle swarm gradually converges to the global best position, and the final parameter combination obtained is the best PSO-Opti-Net parameter setting.

The PSO-Opti-Net for WTB ice cover detection balances global search and local search, allowing the model’s parameter combinations to better fit the data and reduce errors. In this way, the researcher can automatically search the parameter space, which improves the performance of the SP-Y-Net model and provides an effective parameter tuning strategy, which is implemented in the following steps:

1. Initialize the particle swarm: create a swarm of particles, each representing a combination of parameters of the SP-Y-Net model, as follows:

(5) $$P_{i}\;=\;(p_{i1},p_{i2},\ldots ,p_{in})$$

where $P_{i}$ denotes the combination of parameters for particle i and n denotes the number of parameters.

2. Initialize the velocity and position: randomly initialize the velocity and position for each particle as follows:

(6) $$\begin{array}{c} V_{ij}=(v_{ij1},v_{ij2},\ldots ,v_{ijn})\\ X_{ij}=(xi_{j1},x_{ij2},\ldots ,x_{ijn}) \end{array}$$

where $V_{ij}$ and $X_{ij}$ are the velocity and position of the current particle, respectively.

3. Initialize the individual best position: calculate the individual best position of each particle, i.e., find the minimum fitness value in the parameter space as follows:

(7) $$Pbest_{i}=argmin(f(P_{i}))$$

where $argmin\;(f\;(P_{i}))$ represents finding the value of $P_{i}$ that minimizes $f\;(P_{i})$ among all possible values of $P_{i}$.

4. Initialize the global best position: find the particle with the best fitness value in the whole particle swarm and set its parameters as the global best position as follows:

(8) $$Gbest=argmin(f(P_{i}))$$

5. Update Velocity and Position: For each particle, update the velocity and position according to the following equations:

Velocity update:

(9) $$V_{ij}=w\cdot V_{ij}+c_{1}\cdot r_{1}\cdot (Pbest_{ij}-X_{ij})+c_{2}\cdot r_{2}\cdot (Gbest_{j}-X_{ij})$$

Location Updates:

(10) $$X_{ij}=X_{ij}+V_{ij}$$

where $V_{ij}$ denotes the velocity of particle i in dimension j, $X_{ij}$ denotes the current position of particle i in dimension j, ${Pbest}_{ij}$ denotes the individual best position of particle i in dimension j, ${Gbest}_{j}$ denotes the global best position in dimension j, w is the inertia weight, $c_{1}$ and $c_{2}$ are the acceleration factors, and $r_{1}$ and $r_{2}$ are random numbers independently sampled from a uniform distribution in the range [0, 1].

6. Fitness evaluation: For each particle, train the SP-Y-Net model using its current combination of parameters and calculate its prediction error or loss function value as the fitness value as follows:

(11) $$f(P_{i})=LossFuction(P_{i})$$

7. Update Individual Best Position: If the current fitness value of a particle is better than the fitness value of its individual best position, then update the individual best position.

8. Update global best position: If the fitness value corresponding to the individual best position of a particle is better than the fitness value of the global best position, then update the global best position.

9. Iteration: Repeat steps 5 to 8 until the set number of iterations is reached or the convergence condition is reached.

10. Final result: When the PSO converges or reaches the number of iterations, the parameter combination corresponding to the global optimal position finally obtained is the optimal PSO-Opti-Net parameter setting.

## 3. Experiment

### 3.1. Experimental Equipment

#### 3.1.1. Cryogenic Wind Tunnel Experimental System

**Figure 6:**
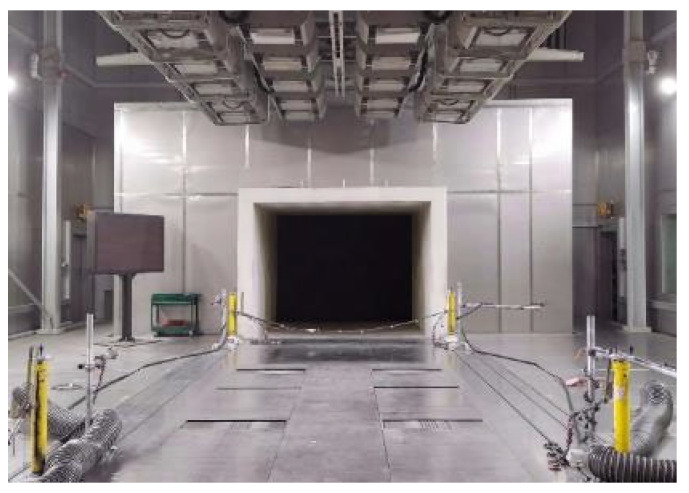
The test section of the environmental wind tunnel laboratory.

Figure 6 shows the test section of the environmental wind tunnel laboratory at the Haval Technology Center. The system utilizes a fan with a total power of 2 MW to provide power for airflow, and the maximum wind speed of the spout can reach 250 km/h. The system realizes temperature regulation through a heat exchanger, which can realize the temperature to be transformed between −40.0 °C~60.0 °C. The laboratory has a super-large-scale spout and test section and contains a water mist injection system, rain and snow simulation system, etc.

#### 3.1.2. Experimental Blade

The experimental blade is produced by Qingdao Yineng Wind Power Equipment Manufacturing Co., Ltd., model YN-2.7 D. The blade is 1400 mm long, made of fiberglass reinforced plastic (FRP), with a rated power of 1 kW, a maximum power of 1.2 kW, an operating wind speed of 3–25 m/s, a rated wind speed of 8 m/s, and a rated rotational speed of 400 r/min. The blade is fixed by a flange connection to a manually adjustable angle of the pedestal.

### 3.2. Experimental Program

After evaluating the preliminary experimental performance and efficiency, and considering the impact of environmental factors on blade icing, an icing type of Glaze ice was selected for its more significant visual effect on ice accumulation. To achieve more visually significant images of ice accumulation on the side of the blade, the blade angle of attack was set to 0°. Considering the icing efficiency, the experimental conditions from group 2 (wind speed 8 m/s) were chosen. The experimental duration was set to 60 min. The specific experimental conditions are presented in Table 1.

### 3.3. Data Preprocessing

Preprocessing operations such as labeling, division, and data enhancement. need to be performed on the images before training the network model. In this paper, the initial data preprocessing includes a normalization process and a wavelet denoising technique to eliminate picture noise, ensuring picture quality and stability. This method will provide clean and consistent data for subsequent prediction.

Normalization is the process of mapping the pixel values of an image to a specific range, usually [0, 1] or [−1, 1], for more efficient analysis. The Min–Max normalization formula is as follows:

(12) $$X_{normalized}=\frac{X-X_{min}}{X_{max}-X_{min}}$$

where X is the original data and $X_{min}$ and $X_{max}$ are the minimum and maximum values of the image pixels, respectively. This formula scales the data to the range [0, 1].

Wavelet denoising is a technique commonly used in signal processing and image noise reduction. It uses wavelet transform to separate the high-frequency and low-frequency components of a signal and then removes the high-frequency noise. The basic equation of the wavelet transform is given below:

(13) $$W(a,b)=\int_{-\infty }^{\infty }x(t)\cdot \psi_{a,b}(t)dt$$

where W(a,b) are the wavelet coefficients, x(t) is the original signal, and $\psi_{a,b}(t)$ is the wavelet basis function, where a and b are the scale and translation parameters. In the technique of wavelet denoising, thresholding is commonly used to eliminate wavelet coefficients that are below a specific threshold to eliminate noise. Once this process of thresholding is performed, an inverse wavelet transform is necessary to regain the denoised signal. The formula for the inverse wavelet transform is mentioned below.

(14) $$x_{denoised}(t)=\sum_{a,b}W_{thresholded}(a,b)\cdot \psi_{a,b}(t)$$

where $x_{denoised}(t)$ is the denoised signal.

**Figure 7:**
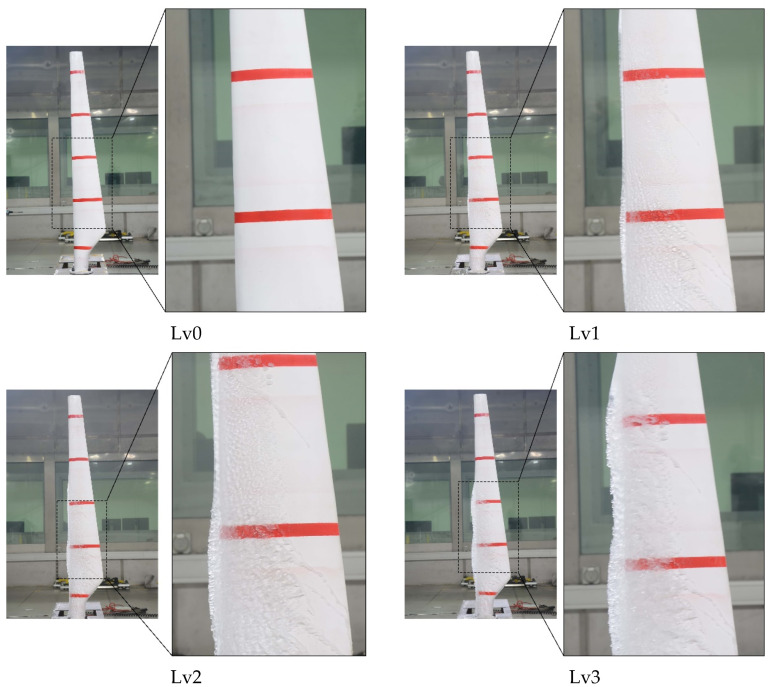
Examples of pictures of WTB icing up at different ratings.

The data preprocessing method of normalization and wavelet denoising is utilized to obtain signals with more prominent features. Subsequently, this paper labels and expands the processed data as follows:

(1) Divide the dataset: To provide a more precise depiction of the various ice-covering conditions, the images captured were categorized during the experiment at 0 min, 20 min, 40 min, and 60 min into four levels: lv0, lv1, lv2, and lv3. This was based on the time at which the experiment was conducted. In this case, ice covering is represented by different levels: lv0 indicates no ice covering, while lv1, lv2, and lv3 correspond to mild, moderate, and severe ice-covering conditions. Sample pictures are displayed in Figure 7. During the screening process, a set of strict quality criteria was applied to the initial 3669 images. Images were discarded if they exhibited significant motion blur, poor focus, over or under-exposure, or contained reflections that obscured the details of ice accretion on the blade surface. Furthermore, only images providing a clear, perpendicular view of the blade’s leading edge were retained to ensure consistency in representing the icing state. This systematic screening process yielded a final high-quality dataset of 3000 images. To ensure the consistency and reliability of the annotations, a standardized labeling protocol was established. Initially, two experienced researchers independently annotated a random subset of 200 images (approximately 7% of the dataset). The annotations were then compared, and any discrepancies were discussed to resolve ambiguities and refine the labeling guidelines. This finalized protocol was subsequently used by one researcher to annotate the entire dataset, with the second researcher performing spot-checks to maintain high-quality standards throughout the process. Among them, there are 806 images belonging to lv0, 730 images belonging to lv1, 694 images of lv2, and 670 images belonging to lv3.
(2) Data annotation: The images of the WTB were labeled utilizing the Labeling tool, wherein the labels corresponded to lv0, lv1, lv2, and lv3, each representing varying degrees of ice coverage. A corresponding XML file is created post-labeling, containing information regarding the size and position of the ice cover as well as the image name and size.
(3) Data Enhancement: Data augmentation techniques are utilized to broaden the dataset, which not only enriches it but also improves the network’s generalization capacity, allowing for better adaptation to various backgrounds in the images. In this study, data augmentation techniques were employed to expand the dataset, thereby enriching its diversity and enhancing the network’s generalization ability. The augmentation strategy adopted herein was strategically designed to improve the model’s robustness against common visual variations encountered in real-world scenarios. Specifically, the dataset was expanded through the application of three key operations: horizontal flipping, Gaussian noise addition, and contrast adjustment. Following data augmentation, the total number of images in the dataset was increased to 10,000, with the training and test sets retaining the original 7:3 proportional split. After data enhancement, the dataset was increased to 10,000 images, with the training and test sets maintaining the original ratio.

### 3.4. Experimental Setup

The initial dataset of 3000 sheets was divided into training and test sets in a 7:3 ratio before data enhancement. After enhancement, the dataset was augmented to 10,000 sheets while maintaining the original ratio. The experimental platform is Python 3, PaddlePaddle 2.3.2, CUDA 11.2, and cuDNN 8.2. The GPU is a Tesla V100, the memory is 16 GB, and the operating system is Linux.

The experiments are conducted in the same computer operating environment. The initial parameters were set as follows: batch size was set to 16, learning rate was 0.01, and the number of training steps was 10,000. Adam optimization was used, and momentum was set to 0.9. Other classical deep learning detection algorithms also used the Adam optimizer for optimization with momentum set to 0.9; the batch size, learning rate, and number of training steps were set according to the network model and data volume.

To achieve the search and optimization of the best model parameters and provide faster convergence, a Particle Swarm Optimization (PSO) algorithm was introduced for model parameter optimization. The core idea of PSO is to establish a particle swarm, where each particle represents a set of parameters for the SP-Y-Net model. These hyperparameters, which govern the training process itself, are optimized using the PSO algorithm. Each particle possesses its own velocity and position, which represent the rate of change of parameters and the current parameter values, respectively. Each particle also maintains a personal best position, corresponding to the parameter setting with the smallest fitness value found by itself, as well as the global best position, which refers to the parameter setting with the smallest fitness value identified by the entire swarm. PSO continuously updates the velocity and position of particles based on these best positions to find better parameter combinations. The process involves multiple iterations until the swarm converges to the global best position. The final parameter combination obtained is the optimal setting for the PSO-Opti-Net model. The fitness function for PSO was defined based on the prediction error or loss function value of the model.

It is worth noting that the optimization problem in this study revolves around improving the performance of the proposed improved PP-YOLOE network for detecting WTB icing status. Specifically, the overarching goal of our study is to obtain a model with optimal performance. This is achieved through two distinct optimization processes: (1) The model’s internal parameters (convolutional layer weights and biases) are optimized through the standard training process via gradient descent. (2) The external training hyperparameters (such as learning rate and batch size), which govern the training process itself, are optimized using the PSO algorithm. The aim of using PSO is to find a hyperparameter configuration that allows the gradient-based training to converge to a better final model. The optimization process is guided by loss functions and training algorithms, which inherently constrain parameter values through gradient descent and other optimization techniques. However, regularization techniques such as weight decay and learning-rate scheduling were adopted to mitigate overfitting and maintain training stability; collectively, these methods impose implicit constraints on the design variables.

Optimization outcomes were assessed by adopting multiple average precision (mAP) at an Intersection over Union (IoU) threshold of 0.5 (mAP@0.5) as the primary evaluation metric. mAP is widely applied in object detection and classification tasks to jointly quantify precision and recall across a range of IoU thresholds. Furthermore, auxiliary indicators such as recall rate, F1 score, mean absolute error, and mean square error were evaluated to provide a comprehensive assessment of model performance. These indicators are reported in detail in the results section to evaluate the effectiveness of our optimization work.

The swarm comprised 30 particles, with a maximum of 50 iterations for convergence. A linearly decreasing inertia weight (w) from 0.9 to 0.4 was employed to balance global exploration and local exploitation. The cognitive and social acceleration factors, c1 and c2, were both set to the standard value of 2.0. The algorithm systematically explored a defined search space for the key training hyperparameters: the initial learning rate was searched within the range [0.0005, 0.01], the batch size was selected from a discrete set of {8, 16, 32}, and the optimizer’s momentum was tuned within the interval [0.85, 0.95].

### 3.5. Ablation Experiments

To assess the effectiveness of the three enhancements of the PSO-Opti-Net network structure, ablation experiments were carried out on each enhancement point of the PP-YOLOE baseline model utilizing the following methodology:

Ablation Experiment 1: Removal of Coordinate Attention Mechanisms

A version without the attention mechanism embedded in the CSPRes module of the backbone network is constructed while keeping other network structures and hyperparameters unchanged. Compare the performance of the models with and without the CA in the task of WTB ice-cover thickness detection, including the metrics Recall, F1-Score, and mAP.

Recall indicates the ability of the model to find all correct objects. A higher rate of find-all indicates that the model can find more positive examples correctly.

(15) $$Recall=\frac{TP}{\left(TP+FN\right)}\times 100\%$$

F1-Score is the reconciled average of Precision and Recall indicators. It is used to balance the Precision and Recall indicators, making the model more stable and not biased toward either the Precision or Recall indicators.

(16) $$F1\_score=2\times \frac{\left(Precision\times Recall\right)}{\left(Precision+Recall\right)}\times 100\%$$

The mAP is used to measure the model performance. It is calculated by averaging multiple average precision (AP) values after summing them. AP is the area under the P-R curve and is used to measure the average accuracy of the model for a single category.

(17) $$\mathrm{mAP}=\frac{1}{k}\sum_{k=1}^{n}AP_{k}$$

where A is the number of positive examples for each category, and $P_{k}$ is interpreted as the predicted probability for the corresponding category

Ablation Experiment 2: Replacing ASPP back to SPP

Replace the ASPP operation in the first CSPBlock module of the middle layer network with the original SPP operation, keeping other network structures and hyperparameters unchanged. Compare the performance of the models using ASPP and SPP on the task of WTB ice-cover thickness detection, including the three metrics of F1-Score, recall, and mAP.

Ablation Experiment 3: Changing parameter settings

The experiment was started using the initial parameter settings as the batch size was set to 16, the learning rate was 0.01, and the number of training steps was 10,000. Adam optimization was used, and the momentum was set to 0.9. Then, the parameters were compared using the parameters that were optimized by the particle swarm, and the comparison parameters were MAE and MSE.

*MAE* (mean absolute error):

(18) $$MAE=\frac{1}{n}\sum_{i=1}^{n}\left|Y_{i}-{\overset{⌢}{Y}}_{i}\right|$$

*MAE* (Mean Squared Error):

(19) $$MSE=\frac{1}{n}\sum_{i=1}^{n}{\left(Y_{i}-{\overset{⌢}{Y}}_{i}\right)}^{2}$$

where n denotes the number of samples, $Y_{i}$ denotes the actual value of the i-th sample, and $\hat{Y_{i}}$ denotes the predicted value of the i-th sample.

## 4. Results and Discussions

### 4.1. Ablation Experiments Results

In ablation experiments, it is necessary to maintain consistency in other parameters and settings to focus only on specific improvements. By comparing the experimental results, the impact of the attention mechanism and ASPP replacement on the PP-YOLOE network in the WTB icing thickness detection task can be evaluated, and their effectiveness and contribution can be determined. Models 1, 2, 3, and 4 represent CSPRes + Attention, CSPRes, CSPBlock + ASPP, and PSO-Opti-Net, respectively. The experimental results are shown in Table 2.

From the results of the ablation experiments, it can be seen that when the training step reaches 10,000, the PSO-Opti-Net network has a recall of 0.9 in the WTB ice-cover thickness detection task, which is 0.03, 0.02, and 0.05 higher than that of CSPRes + Attention, CSPBlock + ASPP, and PP-YOLOE, respectively. The mAP and F1-score are also both higher than those of the other models.

**Figure 8:**
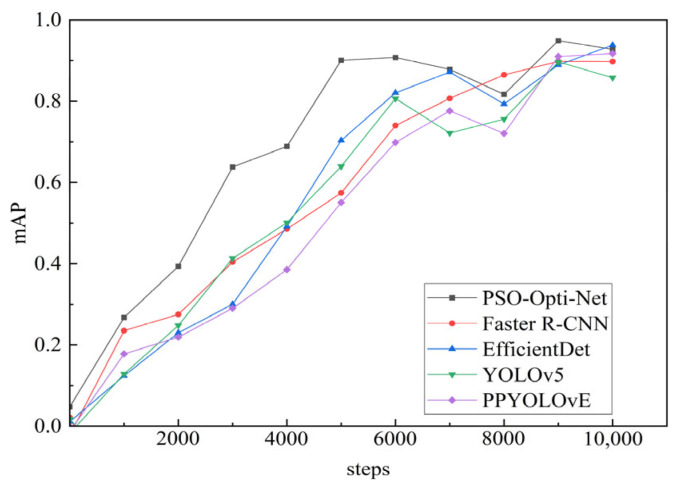
Results of the ablation experiment.

As seen from the results of the ablation experiment in Figure 8, when the training step reaches 10,000, the PSO-Opti-Net network has an accuracy of 0.94 in the WTB ice-cover thickness detection task, which is 0.02, 0.01, and 0.03 higher than CSPRes + Attention, CSPBlock + ASPP and PP-YOLOE, respectively. The detection results achieved a recall of 0.90 across test images, which is also higher than that of CSPRes Attention, CSPBlock ASPP, and PP-YOLOE. It is important to note that while the incremental gains from individual components in the ablation study may appear marginal, their combined effect in the final PSO-Opti-Net model shows a consistent and synergistic improvement across all key metrics (mAP, Recall, and F1-Score). This consistent outperformance suggests that each modification contributes meaningfully to the model’s enhanced capability for this specific task.

The reason for the higher recall and accuracy of CSPRes + Attention over PP-YOLOE is that the attention mechanism helps the model to focus on the regions that are particularly important for the task of identifying ice cover, thus making better use of contextual information. By introducing the attention mechanism, the model can automatically learn which regions are more critical for the identification of ice cover, enabling the model to focus more on these regions and improve the detection of ice cover.

The reason for the higher recall and accuracy of CSPBlock + ASPP over PP-YOLOE is that ASPP can extract features over a different range of receptive fields, including smaller details and larger contextual information. In this way, the model can understand the image content more comprehensively, which helps to detect the ice cover more accurately.

From the results of the ablation experiments, it can be seen that the F1-Score of the PSO-Opti-Net network in the WTB ice cover thickness detection task is 0.92, and the P-R curve of the PSO-Opti-Net network is always above those of CSPRes + Attention, CSPBlock + ASPP and PP-YOLOE. This means that PSO-Opti-Net can provide higher precision at the same recall rate. In other words, the PSO-Opti-Net WTB ice-cover thickness detection task can be recognized more accurately.

The two evaluation metrics, MSE and MAE, are used in ablation experiment 3 in this paper mainly because of their comprehensiveness and usefulness. These two metrics can provide information on multiple aspects of model performance, including the magnitude and direction of errors and robustness to outliers. MSE and MAE are also intuitively interpretable, being able to express the magnitude of errors in the same units as the problem domain, making it easier for decision-makers to understand the results. In addition, these metrics are generalizable across applications, not only for regression problems but also to help compare the performance of different models. Therefore, the selection of MSE and MAE as evaluation metrics helps to comprehensively assess the predictive performance of models and provides a strong basis for model selection and improvement.

In addition, this paper also designs experiments to compare the performance of SP-Y-Net and PSO-Opti-Net in dealing with the prediction of WTB ice-cover thickness, which is used to explore the impact of particle swarm parameter optimization algorithms on the detection task, and the results are shown in Table 3.

Following the optimization of particle swarm parameters, the SP-Y-Net model successfully predicts the ice cover on WTB. This is attributed to the model’s ability to thoroughly optimize parameters. PSO-Opti-Net explores the parameter space to find optimal configurations and prevents the model from encountering a local optimum. As a result, the model adapts better weights and hyperparameter settings to a specific ice cover thickness prediction task. In contrast, an unoptimized SP-Y-Net might use default or empirical parameters that limit the model’s performance. Thus, PSO helps to improve the predictive accuracy and overall performance of the model for superior performance in WTB ice-cover thickness prediction tasks.

### 4.2. Comparison Experiments Results

Various deep learning models have their own unique characteristics and performance attributes. Comparison experiments can analyze their strengths and weaknesses in this particular task and ultimately determine the most appropriate model to deliver precise WTB ice-cover thickness detection results. Comparison experiments can uncover performance variations among different models for detecting the thickness of ice on wind turbine blades. This can serve as a reference point for eventual decision-making and implementation. PPYOLOE, Faster R-CNN, EfficientDet, and YOLOv5 are the selected models for comparison in this paper’s ice-cover thickness detection experiments. The comparison experiments are conducted to evaluate the performance of different models on this task and to select the most suitable model for application. The experimental results are shown in Table 4.

Based on the results of the comparison experiments, it is evident that when the training stage reaches 10,000, the PSO-Opti-Net network has a recall rate of 0.9 in identifying the thickness of the ice covering wind blades. This recall rate is higher than that of PPYOLOE and Faster R-CNN, and comparable to the performance level of the YOLOv5 model, while being slightly lower than EfficientDet’s 0.91.

**Figure 9:**
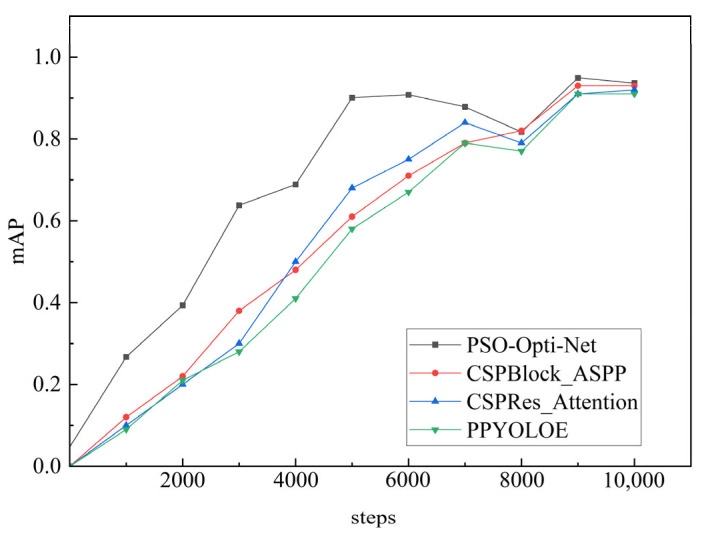
Comparison of average accuracy curves.

As seen from the results of the comparison experiments in Figure 9, when the training step reaches 10,000, the PSO-Opti-Net network has an average accuracy of 0.94 in the task of detecting the thickness of WTB ice cover, which is higher than that of PPYOLOE, Faster R-CNN, EfficientDet, and YOLOv5. Considering that PSO-Opti-Net uses the PANet structure, the structure can effectively fuse feature information from different scales. This makes PSO-Opti-Net more advantageous in dealing with targets of different sizes and therefore better than models such as YOLOv5 for WTB ice-cover thickness detection.

Based on the comparison experiments, it is evident that the P-R curve of the PSO-Opti-Net network consistently outperforms that of PPYOLOE, Faster R-CNN, EfficientDet, and YOLOv5. This indicates that the proposed model in this paper operates optimally.

The outstanding performance of PSO-Opti-Net in recall, accuracy, and F1-Score is attributed to the application of the attention mechanism and ASPP, optimization of the model structure, and enhanced data and training procedures. These factors collectively contribute to the exceptional performance of the model. By implementing these enhancements, PSO-Opti-Net becomes capable of more precise recognition of the attributes concerning the accumulation of ice on WTB, enhancing the aptitude for identifying the magnitude of this accumulation and surpassing other approaches in terms of evaluation metrics.

### 4.3. Detection Effect

In this study, the optimization problem revolves around enhancing the performance of the proposed improved PP-YOLOE network for detecting icing states of WTBs. Specifically, model parameters, including but not limited to convolutional layer weights and biases are optimized to minimize classification error, thereby enhancing overall accuracy. This optimization is implicitly addressed through the training process, where the model is trained to minimize a loss function that reflects the classification error.

**Figure 10:**
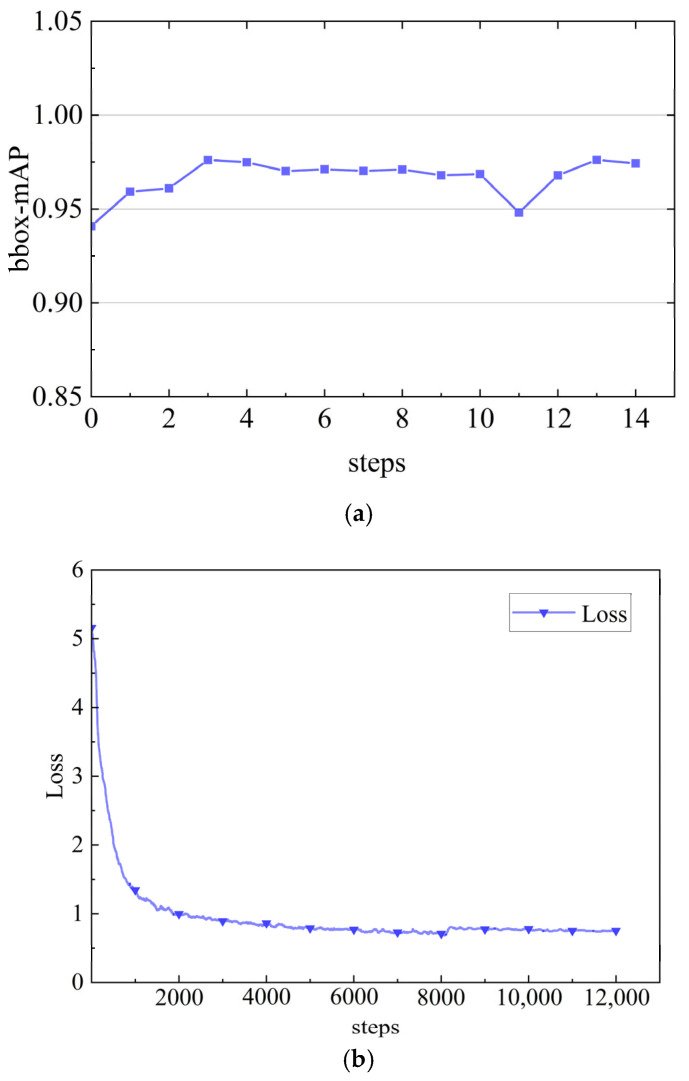
Visualization of model training. (**a**) The mAP value change curve. (**b**) Loss value change curve.

In Figure 10, the process of training the model is visualized. Our experiments have shown that mAP has reached a relatively high level and remains stable over multiple iterations. The stability of mAP on the validation set suggests that the model has effectively learned the features within our dataset and converged well, without showing signs of significant overfitting to the training data. The fact that mAP has not undergone significant changes beyond this point is a powerful indicator of convergence. Similarly, the loss function measures the difference between the predicted label and the true label and converges to a stable value after 10,000 iterations. The loss curve tends to stabilize, indicating that the model no longer shows significant improvement in reducing prediction errors. This is a sign of convergence in machine learning models. The model checkpoint with the highest validation mAP of 0.976 was saved during training. For final evaluation on the test set, this model achieved a stable and generalizable mAP of 0.94, which is the value reported in our comparative results.

**Figure 11:**
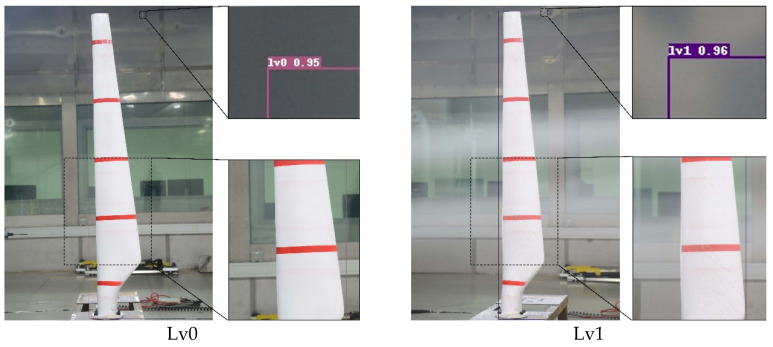

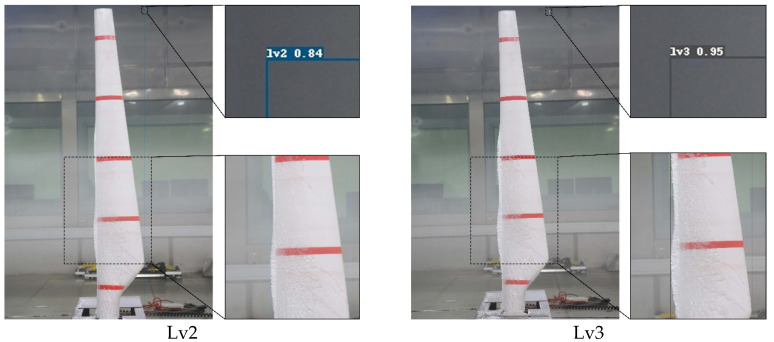
PSO-Opti-Net detection effect diagram.

In the validation of wind turbine blade ice-covered images using the optimal neural network training model, Figure 11 illustrates the experimental impact.

While this study demonstrates the effectiveness of the proposed PSO-Opti-Net model, this study acknowledges certain limitations that lay the groundwork for future research. A primary limitation stems from the dataset being collected within a controlled wind tunnel laboratory, utilizing a single blade model under fixed environmental parameters and a static angle of attack. This controlled setting, while crucial for foundational validation, may not fully capture the complexities of real-world operational environments, which involve dynamic blade rotation, diverse weather patterns, variable lighting, and different blade types. Consequently, the model’s generalization capability across these varied, uncontrolled scenarios warrants further investigation.

## 5. Conclusions

To detect WTB ice covering, an improved PP-YOLOE network, PSO-Opti-Net, is proposed in this paper. After experimental testing, PSO-Opti-Net outperformed PP-YOLOv2, Faster R-CNN, EfficientDet, and YOLOv5 in three aspects: recall, accuracy, and F1-Score. PSO-Opti-Net has a Recall of 0.90, compared to the other models’ Recall scores of 0.85 (PP-YOLOE), 0.88 (Faster R-CNN), 0.91 (EfficientDet), and 0.90 (YOLOv5). PSO-Opti-Net achieves a mAP of 0.94, while the other models have mAP scores of 0.91 (PP-YOLOE), 0.93 (Faster R-CNN), 0.92 (EfficientDet), and 0.93 (YOLOv5). This shows that PSO-Opti-Net leads the other models in terms of the average accuracy of detection results. In addition, PSO-Opti-Net’s F1 score is 0.92, while PP-YOLOE’s F1 score is 0.87, Faster R-CNN’s F1 score is 0.91, EfficientDet’s F1 score is 0.90, and YOLOv5’s F1 score is 0.91, which reflects that PSO-Opti-Net has a good balance between precision and recall rate is well balanced and performs better when accuracy and recall are combined.

The performance of model is driven by key innovations, including architectural enhancements through the attention mechanism and ASPP, and a novel ap-plication of particle swarm optimization for systematic hyperparameter finding. These adjustments enable the model to capture the characteristics of wind blade ice cover more accurately. Compared to previous methods used to detect icing on wind turbine blades, PSO-Opti-Net bases its prediction on global image information, as opposed to detection algorithms that employ sliding windows as well as area suggestions. Compared to two-stage detectors like Faster R-CNN, which achieved a mAP of 0.93, our single-stage PSO-Opti-Net not only achieved a higher mAP of 0.94 but also demonstrated strong performance in extracting generic and representational features of the object directly from the global image information. This contributes to its good performance and robustness on the established dataset.

The wind turbine blade icing detection model based on PP-YOLOE network introduced in this article improves the balance between detection accuracy and speed, and reduces dependence on anchor point settings compared to some traditional architectures, and avoids special operators such as deformable convolution and matrix NMS (Non-Maximum Suppression), making it easy to deploy on a wide range of hardware and providing guidance for improving the accuracy of intelligent detection and recognition of wind turbine blades. Ultimately, this research not only provides an efficient tool for WTB ice detection, but also demonstrates a referenceable approach that can integrate deep learning components to address more complex detection challenges.

Future work will focus on extending the dataset to include images from in-service wind turbines, thereby enhancing the model’s robustness and applicability. To further improve performance in complex visual environments, plans are outlined to explore advanced signal processing methods for image quality enhancement, such as the utilization of Positive-Incentive noise concepts—concepts that have demonstrated promise in fault diagnosis. Moreover, addressing the inherent “black-box” nature of deep learning constitutes a critical future direction, with the aim of enhancing the model’s interpretability to foster greater trust for industrial deployment. This objective will draw inspiration from emerging techniques that improve the transparency of AI decisions. Collectively, these efforts will advance the development of a more reliable and field-deployable intelligent system for WTB icing detection.

## Figures and Tables

**Table 1:** Experimental conditions.

Experimental Conditions		Values
Temperature T	$\left({}^{\circ}C\right)$	−6
Wind speed U	$\left(m\cdot s^{-1}\right)$	8
Angle α	(°)	0
Experimental time t	(min)	60
Shooting interval Δt	(min)	20

**Table 2:** Ablation study with different component combinations on the dataset. The use of bold is the identification of key data.

Model	1	2	3	4	Recall	mAP	F1
Exp 1	-	√	-	√	0.85	0.91	0.87
Exp 2	-	√	√	-	0.88	0.93	0.90
Exp 3	√	-	-	√	0.87	0.92	0.89
Exp 4	√	-	√	-	**0.90**	**0.94**	**0.92**

**Table 3:** Error comparison between SP-Y-Net and PSO-Opti-Net in WTB ice detection.

Model	MSE	MAE
PSO-Opti-Net	58.412	0.921
SP-Y-Net	70.836	1.003

**Table 4:** Comparative experimental results with SOTA methods. The use of bold is the identification of key data.

Model	Recall	mAP	F1
PSO-Opti-Net	**0.90**	**0.94**	**0.92**
PP-YOLOE	0.85	0.91	0.87
Faster R-CNN	0.88	0.93	0.91
EfficientDet	0.91	0.92	0.90
YOLOv5	**0.90**	0.93	0.91

## Data Availability

The original contributions presented in this study are included in the article. Further inquiries can be directed to the corresponding author.

## Abbreviation

**Abbreviation**	**Full Name**	**Description/Field**
**PP-YOLOE**	-	Excellent single-stage anchor-free object detection model based on PP-YOLOv2
**WTB**	Wind Turbine Blades	Converting wind energy into mechanical power through aerodynamic design
**CA**	Coordinate Attention	An attention mechanism that enhances feature representation by modeling channel relationships and positional information simultaneously
**CSPBlock**	Cross Stage Partial Block	A neural network block design that splits features across stages and fuses them partially
**SPP**	Spatial Pyramid Pooling	A pooling strategy that enables CNNs to accept variable-sized inputs by generating fixed-length feature vectors
**PANet**	Path Aggregation Network	A neural network architecture that enhances information flow and feature representation by aggregating features across different layers and scales
**ASPP**	Atrous Spatial Pyramid Pooling	A pooling method that utilizes atrous convolutions at multiple sampling rates to capture multi-scale contextual information
**PSO**	Particle Swarm Optimization	An optimization algorithm based on swarm intelligence
**FRP**	Fiberglass Reinforced Plastics	A composite material consisting of a plastic matrix reinforced with glass fibers
**MAE**	Mean Absolute Error	A metric that measures the average magnitude of the errors in a set of predictions, without considering their directions
**MSE**	Mean Squared Error	A metric that measures the average squared difference between the predicted and actual values, giving higher weights to larger errors
**Y-Att-Net**	-	Introduction of measurement model based on PP-YOLOE attention mechanism
**SP-Y-Net**	-	A model established by combining Y-Att-Net with ASPP structure
**PSO-Opti-Net**	-	Introducing particle swarm optimization to fine tune the parameters of the SP-Y-Net model
**TN**	True Negative	The four metrics serve as fundamental building blocks for evaluating the performance of classification models, quantifying their ability to correctly and incorrectly identify positive and negative instances
**TP**	True Positive	
**FN**	False Negative	
**FP**	False Positive	
**AP**	average precision	A metric that measures the average precision of a model at all recall levels
**mAP**	multiple average precision	The mean of AP scores across multiple classes or queries
**F1-Score**	-	The harmonic means of precision and recall, used as a metric to evaluate the performance of classification models
**Recall**	-	The proportion of relevant instances that are correctly retrieved by a model out of the total number of relevant instances present in the dataset
**CNN**	Convolutional Neural Networks	A type of deep learning algorithm that mimics the structure and function of the human visual system
**EfficientDet**	Scalable and Efficient Object Detection	A scalable and efficient detection model utilizing Efficient Net scaling and Bifan for multi-scale feature fusion
**Faster R-CNN**	Faster Region-based Convolutional Neural Network	A two-stage object detection network using CNN for classification and bounding box regression
**YOLOv5**	-	The fifth iteration of the YOLO (You Only Look Once) real-time object detection system
**NMS**	Non-Maximum Suppression	A technique used to suppress non-maximum values, enhancing the detection of local maxima in tasks such as object detection and edge detection

## References

[1] IRENA, ILO (2023). Renewable Energy and Jobs: Annual Review 2023.

[2] Farina A., Anctil A. (2022). Material consumption and environmental impact of wind turbines in the USA and globally. Resour. Conserv. Recycl..

[3] Sundaresan A., Arunvinthan S., Pasha A.A., Nadaraja Pillai S. (2021). Effect of ice accretion on the aerodynamic characteristics of wind turbine blades. Wind Struct..

[4] Lamraoui F., Fortin G., Benoit R., Perron J., Masson C. (2014). Atmospheric icing impact on wind turbine production. Cold Reg. Sci. Technol..

[5] Fang Y., Zhao S. (2020). Joint Optimal Operation and bidding strategies of concentrating solar power plants with wind farms. Proc. CSEE.

[6] Shajiee S., Pao L.Y., Wagner P.N., Moore E.D., McLeod R.R. Direct Ice Sensing and Localized Closed-Loop Heating for Active Deicing of Wind Turbine Blades. Proceedings of the 2013 American Control Conference.

[7] Roberge P., Lemay J., Ruel J., Bégin-DroletShow A. (2023). Understanding ice accretion on wind turbines with field data. Cold Reg. Sci. Technol..

[8] Zhang Y., Zhao P., Chi H., Guo W., Li Y. (2024). Impact of icing on the flow field of wind turbine blades with different airfoils. Vortex Workshop.

[9] Jin J.Y., Virk M.S. (2020). Experimental study of ice accretion on S826 & S832 wind turbine blade profiles. Cold Reg. Sci. Technol..

[10] Yang X.Y., Zhang Y.F., Lv W., Wang D. (2021). Image recognition of wind turbine blade damage based on a deep learning model with transfer learning and an ensemble learning classifier. Renew. Energy.

[11] Yirtici O., Tuncer I.H. (2021). Aerodynamic shape optimization of wind turbine blades for minimizing power production losses due to icing. Cold Reg. Sci. Technol..

[12] Gao L.Y., Liu Y., Zhou W.W., Hu H. (2019). An experimental study on the aerodynamic performance degradation of a wind turbine blade model induced by ice accretion process. Renew. Energy.

[13] Gao L.Y., Veerakumar R., Liu Y., Hu H. (2019). Quantification of the 3D shapes of the ice structures accreted on a wind turbine airfoil model. J. Vis..

[14] Gao L.Y., Liu Y., Hu H. (2020). An experimental investigation on the dynamic glaze ice accretion process over a wind turbine airfoil surface. Int. J. Heat Mass Transf..

[15] Gao Y.Y., Liu Y., Hu H. (2019). An experimental investigation of dynamic ice accretion process on a wind turbine airfoil model considering various icing conditions. Int. J. Heat Mass Transf..

[16] Afzal F., Virk M.S., Bevrani H. (2018). Review of Icing Effects on Wind Turbine in Cold Regions. E3S Web Conf..

[17] Frohboese P., Anders A. (2007). Effects of Icing on Wind Turbine Fatigue Loads. J. Phys. Conf. Ser..

[18] Homola M.C., Virk M.S., Nicklasson P.J., Sundsbø P.A. (2012). Performance losses due to ice accretion for a 5 MW wind turbine. Wind. Energy.

[19] Wang W.J., Xue Y., He C.K., Zhao Y. (2022). Review of the typical damage and damage-detection methods of large wind turbine blades. Energies.

[20] Tammelin B., Böhringer A., Cavaliere M., Holttinen H., Morgan C., Seifert H., Säntti K., Vølund P. (2000). Wind Energy Production in Cold Climate (WECO).

[21] Du Y., Zhou S., Jing X., Peng Y., Wu H., Kwok N. (2020). Damage mitigation techniques in wind turbine blades: A review. Mech. Syst. Signal Process..

[22] Battisti L. (2011). Optimizing wind turbine design for operation in cold climates. Wind Energy Systems.

[23] Bégin-Drolet A., Ruel J., Lemay J., Giroux G. (2012). Commissioning of a new ice-free anemometer: 2011 Field tests at WEICan. Measurement.

[24] Hacefendiolu K., Başağa H.B., Yavuz Z., Karimi M.T. (2022). Intelligent ice detection on wind turbine blades using semantic segmentation and class activation map approaches based on deep learning method. Renew. Energy.

[25] Sun S.L., Li Q., Hu W.Y., Liang Z., Wang T., Chu F. (2023). Wind turbine blade breakage detection based on environment-adapted contrastive learning. Renew. Energy.

[26] Zhang X., Ding Y., Zhao H., Yi L., Guo T., Li A. (2024). Mixed Skewness Probability Modeling and Extreme Value Predicting for Physical System Input–Output Based on Full Bayesian Generalized Maximum-Likelihood Estimation. IEEE Trans. Instrum. Meas..

[27] Badihi H., Zhang Y., Jiang B., Pillay P., Rakheja S. (2022). A Comprehensive Review on Signal-Based and Model-Based Condition Monitoring of Wind Turbines: Fault Diagnosis and Lifetime Prognosis. Proc. IEEE.

[28] Li D., Ho M.S.-C., Song G., Ren L., Li H. (2015). A review of damage detection methods for wind turbine blades. Smart Mater. Struct..

[29] Hyers R.W., Mcgowan J.G., Sullivan K.L., Manwell J.F., Syrett B.C. (2006). Condition monitoring and prognosis of utility scale wind turbines. Energy Mater..

[30] Wiggelinkhuizen E., Verbruggen T., Braam H., Rademakers L., Xiang J., Watson S. (2008). Assessment of Condition Monitoring Techniques for Offshore Wind Farms. ASME J. Sol. Energy Eng..

[31] Hu Q., Xu X., Leng D., Shu L., Jiang X., Virk M., Yin P. (2021). A Method for Measuring Ice Thickness of Wind Turbine Blades Based on Edge Detection. Cold Reg. Sci. Technol..

[32] Akhloufi M.A., Benmesbah N. (2014). Outdoor Ice Accretion Estimation of Wind Turbine Blades Using Computer Vision. Proceedings of the 2014 Canadian Conference on Computer and Robot Vision (CRV).

[33] Skrimpas G.A., Kleani K., Mijatovic N., Sweeney C.W., Jensen B.B., Holbøll J. (2016). Detection of icing on wind turbine blades by means of vibration and power curve analysis. Wind. Energy.

[34] Yang C., Qiao Z., Liu L., Kumar A., Zhu R. (2025). Positive-incentive noise in artificial intelligence-enabled machine fault diagnosis. Struct. Health Monit..

[35] Zhao H., Zhang X., Ding Y., Guo T., Li A., Soh C.K. (2025). Probabilistic mixture model driven interpretable modeling, clustering, and predicting for physical system data. Eng. Appl. Artif. Intell..

[36] Zhang C., Yang T., Yang J. (2022). Image Recognition of Wind Turbine Blade Defects Using Attention-Based MobileNetv1-YOLOv4 and Transfer Learning. Sensors.

[37] Yao Y., Wang G., Fan J. (2023). WT-YOLOX: An Efficient Detection Algorithm for Wind Turbine Blade Damage Based on YOLOX. Energies.

[38] Gao R.X., Ma Y.F., Wang T.F. (2023). Early stage damage detection of wind turbine blades based on UAV images and deep learning. J. Renew. Sustain. Energy.

[39] Kreutz M., Alla A.A., Eisenstadt A., Freitag M., Thoben K.-D. (2020). Ice Detection on Rotor Blades of Wind Turbines using RGB Images and Convolutional Neural Networks. Procedia CIRP.

[40] Xu S., Wang X., Lv W., Chang Q., Cui C., Deng K., Wang G., Dang Q., Wei S., Du Y. (2022). PP-YOLOE: An evolved version of YOLO. arXiv.

[41] Jafari M., Salajegheh E., Salajegheh J. (2021). Optimal design of truss structures using a hybrid method based on particle swarm optimizer and cultural algorithm. Structures.

[42] Qolomany B., Maabreh M., Al-Fuqaha A., Gupta A., Benhaddou D. Parameters optimization of deep learning models using Particle swarm optimization. Proceedings of the 2017 13th International Wireless Communications and Mobile Computing Conference (IWCMC).

[43] Hou Q., Zhou D., Feng J. Coordinate attention for efficient mobile network design. Proceedings of the 2021 IEEE/CVF Conference on Computer Vision and Pattern Recognition (CVPR).

[44] Chen L.C., Papandreou G., Schroff F., Adam H. (2017). Rethinking atrous convolution for semantic image segmentation. arXiv.

